# Comparison of factors influencing fall recurrence in the young-old and old-old: a cross-sectional nationwide study in South Korea

**DOI:** 10.1186/s12877-022-03172-7

**Published:** 2022-06-25

**Authors:** Mi Young Kim, Yujeong Kim

**Affiliations:** 1 College of Nursing, Hanyang University, 222 Wangsimni-ro, Seongdong-gu, Seoul, 04763 Republic of Korea; 2 College of Nursing, Research Institute of Nursing Science, Kyungpook National University, 680 Gukchabosangro, Jung-gu, Daegu, 41944 Republic of Korea

**Keywords:** Accidental falls, Aged, Frail elderly, Old-old, Young-old

## Abstract

**Background:**

Recurrent falls are a concerning problem in the elderly. Elderly people aged > 65 years who are prone to fall often require medical treatment for severe fall-related injuries, which is associated with a substantial financial burden. Therefore, this study aimed to identify factors related to recurrent falls in the community-dwelling young-old (65–74 years old) and old-old (≥ 75 years) in South Korea.

**Methods:**

This study used a cross-sectional, correlation design. Data from the 2017 National Survey of Older Koreans were used, and 5,838 young-old and 4,205 old-old elderly people were included in the analysis. The questionnaire included general characteristics, fall experience, physical status, mental status, and presence of chronic diseases. The data were analyzed using the chi-square test, one-way analysis of variance, and logistic regression analysis.

**Results:**

In the young-old elderly people, limitations in activities of daily living (*p* < .001), use of visual aids (*p* = .002), cognitive function (*p* < .001), presence of suicidal ideations (*p* = .005), number of chronic diseases (*p* < .001), and number of prescribed medications used (*p* = .006) associated with fall recurrence. In the old-old elderly people, having a spouse (*p* = .034), being a beneficiary of the National Basic Livelihood Security System (*p* = .025), less exercise (*p* = .003), limitations in activities of daily living (*p* < .001), visual aid use (*p* = .002), presence of suicidal ideations (*p* = .015), number of chronic diseases (*p* < .001), and presence of Parkinson's disease (*p* < .001) associated with fall recurrence.

**Conclusions:**

This study identified differences in factors related to fall recurrence between the young-old and old-old elderly. The results of this study indicate that it is necessary to implement an intervention program to prevent fall recurrence by age group in consideration of the risk factors for fall recurrence in each elderly people group.

**Supplementary Information:**

The online version contains supplementary material available at 10.1186/s12877-022-03172-7.

## Background

In Korea, 15.7% of the total population of 8,125,000 people is older than 65 years and because Korea is a super-aged society, this proportion is expected to increase to 20.3% by 2025 [1]. According to the United Nations, a super-aged society refers to a society where more than 20% of the total population is aged 65 years and older. Physical activity and mobility tend to decline gradually with aging due to decline in physical function [2]. Elderly people comprise a vulnerable age group with multiple health problems that threaten their safety and quality of life. According to the World Health Organization (WHO), falls are a major public health problem worldwide [3]. Approximately 646,000 fatal falls occur annually, with falls being the second leading cause of death from unintended injuries, after road traffic injuries [3]. In an aged society, elderly peoples’ falls are a very concerning problem. Most of elderly people experience fall injuries, and severe fractures have been observed in 5%–10% of those elderly people [4]. In particular, the elderly, over the age of 65 years, with fall episodes often require medical treatment for severe fall-related injuries, causing considerable financial burden on the patients and their families [3].

To prevent falls, studies have evaluated various related predictive factors. The risk factors for falls that have been reported in different studies include age [5], sex [6], income level, and having a spouse [7]. Additionally, muscle strength, balance ability, sensory deficits [8], number of chronic diseases [5], presence of comorbid conditions, and use of certain categories of medications [8] have also been reported as risk factors.

Falls may occur repeatedly in individuals. In fact, 50% of elderly people population have experienced recurrent falls [9]. Those who have experienced fall recurrence have been observed to have greater morbidity than those who did not [10]. Thus, the consequences of recurrent falls are serious. However, there is a lack of studies on the recurrence of falls. As reported in previous studies, single and recurrent falls have different mechanisms [11]. While single falls may result from accidents including environmental hazards and overwhelming incidences such as myocardial infarction, recurrent falls are more likely to be caused by intrinsic factors [12]. Therefore, it may be necessary to assess factors that affect fall recurrence.

In studies on the elderly, individuals with age differences of 20–30 years have been grouped together for comprehensive assessment; however, such a classification may overlook the differences in health conditions and the problems of the young-old (65–74 years old) and old-old (≥ 75 years). [13]. Therefore, Neugarten et al. [14] suggested dividing elderly people into the young-old and old-old groups under and over the age of 75 years, respectively. In fact, the mean cost of health care for the old-old elderly people has been shown to be almost twice as much as that for the young-old elderly. The old-old elderly people have also been reported to be physically, mentally, and financially more vulnerable than the young-old elderly people [15].

To predict and prevent fall recurrence in the elderly, we must understand the risk factors for recurrent falls and identify the predictive factors of falls with detailed standards that consider the characteristics of elderly people by age group. Therefore, this study assessed the predictive factors of fall recurrence in the young-old and old-old elderly people.

## Methods

### Study sample

This cross-sectional study aimed to identify the factors that are related to recurrent falls in the young-old and old-old elderly. This study used raw data from the 2017 National Survey of Older Koreans that was conducted by the Korea Institute for Health and Social Affairs using interviews of individuals aged over 65 years living in community. Random sampling was performed by stratifying the population, and subjects living in special care facilities were excluded. Among the 10,299 participants aged over 65 years, those with missing values in related measured items were excluded, and 5,838 and 4,205 young-old and old-old elderly people were analyzed in this study.

### Measures

#### Fall experience

The item “Have you experienced falls (falling, slipping, or collapsing) in the past year? If yes, how many times?” was used to assess fall experience and was answered as yes, no, and the number of times, if any.

#### General characteristics

Sex, education level, having a spouse, National Basic Livelihood Security System (NBLSS) beneficiaries, smoking habit, problematic drinking, and exercise were assessed. The NBLSS is a government-supported benefit to guarantee minimum living and is indicative of poverty. The current smoking status was evaluated, and problematic drinking was defined as the consumption of more than three drinks daily in the last month. Exercise was assessed based on the responses “yes” or “no” to exercising more than once a week for 10 min or more each time.

#### Physical status

Factors including limitations in activities of daily living (ADL) and instrumental activities of daily living (IADL), visual aids, and body mass index (BMI) were measured to evaluate the participants’ physical status. ADL were measured using the Korean version of the daily life activity measurement tool [16]. Further, complete independence, partial dependence, and complete dependence on seven items in the last 7 days were assessed, and partial or complete dependence for one or more items were considered limitations in ADL. IADL were measured using the Korean version of the IDAL (K-IADL) measurement tool [16]. Complete independence, partial dependence, and complete dependence on 10 items and partial and complete dependence for one or more items were considered limitations in IADL. ADL assess the minimal ability of elderly people to live independently (e.g., eating, toileting, and bathing), whereas IADL measure their ability to respond to social circumstances (e.g., public transport, shopping, housework, and medication) [16]. In our study, the Cronbach’s alpha values for the K-ADL and K-IADL were 0.78 and 0.89, respectively. Vision aid was assessed by determining whether elderly people used glasses or contact lenses, and BMI was calculated using height and weight.

#### Mental status

Mental status including depression and suicidal ideation were measured by self-report questionnaire. Depression was assessed based on whether the participants experienced depression for more than 3 months, and suicidal ideation was assessed based on the answers to the item “Have you ever considered committing a suicide after the age of 60?” (yes or no).

#### Chronic disease characteristics

The chronic disease characteristics were assessed according to cognitive function of the participants and the number of chronic diseases present for more than 3 months (according to the doctor's diagnosis). The cognitive function was measured using the Korean Version of the Mini Mental Status Examination for Dementia Screening (MMSE-DS) [17]. The MMSE-DS contains 19 items for a total of 30 points. The Cronbach’s alpha was 0.83 in a study by Kim et al. [17] and 0.81 in this study. Chronic diseases included hypertension, stroke, angina/myocardial infarction, diabetes, arthritis, osteoporosis, lumbodynia, sciatic neuralgia, cataract, glaucoma, cancer, chronic kidney disease, prostatic hypertrophy, urinary incontinence, anemia, dementia, insomnia, and Parkinson's disease. The number of prescribed medications taken daily for more than 3 months was also assessed.

### Statistical analysis

Statistical analyses were performed using IBM SPSS 25.0. The participants’ general characteristics, physical and mental states, and chronic disease characteristics are presented as frequency, percentage, mean, and standard deviation. The differences in fall frequency were analyzed according to the participants’ general characteristics, physical and mental states, and chronic disease characteristics using the chi-square test, one-way analysis of variance, and Scheffe test for post-hoc comparisons. The factors affecting recurrent falls were analyzed using a logistic regression analysis.

## Results

### Differences in fall frequency according to general characteristics

Single falls occurred in 516 (8.8%) and 516 (12.3%) participants in the young-old and old-old groups, respectively, whereas recurrent falls occurred in 263 (4.5%) and 283 (6.7%) participants, respectively.

In the young-old group, recurrent falls rates were significantly associated with females, those with education level below elementary school, those with no spouse, those who were NBLSS beneficiaries, those who did not smoke, those who were not problematic drinkers, and those who exercised. In the old-old group, recurrent falls rates were significantly associated with females, those who had no spouse, those who were NBLSS beneficiaries, and those who did not exercise (Table 1).

**Table 1:** Differences in fall frequency according to general characteristics (*N* = 10,043)

Variables	Category	Young-old (*n* = 5,838)				Old-old (*n* = 4,205)			
		No fall (*n* = 5,060)	Single fall (*n* = 516)	Recurrent fall (*n* = 263)	Χ^2^, F(*P*) Scheffe	No fall (*n* = 3,406)	Single fall (*n* = 516)	Recurrent fall (*n* = 283)	Χ^2^, F(*P*) Scheffe
Sex	Male	2,303 (91.1)	137 (5.4)	89 (3.5)	78.17 (< .001)	1,499 (86.0)	158 (9.1)	86 (4.9)	48.08 (< .001)
	Female	2,757 (83.3)	378 (11.4)	174 (5.3)		1,907 (77.5)	358 (14.5)	196 (8.0)	
Education level	≤ Elementary school	2,506 (84.6)	295 (10.0)	162 (5.5)	25.87 (< .001)	2,271 (79.4)	380 (13.3)	209 (7.3)	17.01 (.009)
	Middle school	1,077 (88.6)	97 (8.0)	41 (3.4)		414 (83.8)	50 (10.1)	30 (6.1)	
	High school	1,075 (88.4)	91 (7.5)	50 (4.1)		444 (83.6)	58 (10.9)	29 (5.5)	
	≥ College	402 (90.1)	33 (7.4)	11 (2.5)		277 (86.8)	28 (8.8)	14 (4.4)	
Having a spouse	No	1,324 (82.1)	201 (12.5)	87 (5.4)	42.40 (< .001)	1,546 (76.2)	318 (15.7)	165 (8.1)	59.49 (< .001)
	Yes	3,735 (88.4)	315 (7.5)	176 (4.2)		1,859 (85.5)	198 (9.1)	118 (5.4)	
NBLSS^a^ beneficiary	No	4,779 (87.1)	468 (8.5)	241 (4.4)	14.35 (.001)	3,160 (81.7)	468 (12.1)	240 (6.2)	23.85 (< .001)
	Yes	281 (80.1)	48 (13.7)	22 (6.3)		246 (73.0)	48 (14.2)	43 (12.8)	
Smoking habit	No	4,414 (86.1)	479 (9.3)	234 (4.6)	13.87 (.001)	3,148 (80.9)	478 (12.3)	263 (6.8)	0.28 (.870)
	Yes	646 (90.6)	37 (5.2)	30 (4.2)		258 (81.9)	38 (12.1)	19 (6.0)	
Problematic drinking	No	4,766 (86.2)	504 (9.1)	256 (4.6)	15.16 (.001)	3,277 (80.9)	500 (12.3)	275 (6.8)	1.10 (.577)
	Yes	294 (93.9)	12 (3.8)	7 (2.2)		128 (84.2)	16 (10.5)	8 (5.3)	
Exercise	No	1,369 (84.7)	164 (10.1)	84 (5.2)	7.70 (.021)	1,237 (77.8)	209 (13.1)	144 (9.1)	25.39 (< .001)
	Yes	3,691 (87.4)	351 (8.3)	180 (4.3)		2,169 (82.9)	307 (11.7)	139 (5.3)	

^a^*NBLSS* National Basic Livelihood Security System

### Differences in fall frequency according to physical and mental states

In the young-old group, recurrent falls rates were significantly associated with limitations in ADL or IADL, used visual aids, had a high BMI, had depression, and had suicidal ideation. In the old-old group, recurrent falls rates were significantly associated with limitations in ADL or IADL, used visual aids, had depression, and had suicidal ideation (Table 2).

**Table 2:** Differences in fall frequency according to physical and mental states (*N* = 10,043)

Variables	Category	Young-old (*n* = 5,838)				Old-old (*n* = 4,205)			
		No fall (*n* = 5,060)	Single fall (*n* = 516)	Recurrent fall (*n* = 263)	Χ^2^, F(*P*) Scheffe	No fall (*n* = 3,406)	Single fall (*n* = 516)	Recurrent fall (*n* = 283)	Χ^2^, F(*P*) Scheffe
Limitations in ADL^a^	No	4,938 (87.3)	482 (8.5)	234 (4.1)	82.08 (< .001)	3,063 (82.8)	428 (11.6)	207 (5.6)	81.62 (< .001)
	Yes	122 (65.9)	34 (18.4)	29 (15.7)		342 (67.7)	88 (17.4)	75 (14.9)	
Limitations in IADL^b^	No	4,526 (88.0)	424 (8.2)	193 (3.8)	82.78 (< .001)	2,235 (85.3)	255 (9.7)	129 (4.9)	86.10 (< .001)
	Yes	533 (76.6)	92 (13.2)	71 (10.2)		1,171 (73.8)	261 (16.5)	154 (9.7)	
Visual aid	No	1,959 (88.4)	183 (8.3)	75 (3.4)	12.55 (.002)	1,337 (83.5)	180 (11.2)	84 (5.2)	12.69 (.002)
	Yes	3,101 (85.6)	333 (9.2)	188 (5.2)		2,069 (79.5)	336 (12.9)	199 (7.6)	
BMI^c^		23.91 ± 2.93^a^	24.29 ± 3.18^b^	24.20 ± 3.37^c^	4.80 (.008) a < b	23.06 ± 3.06	23.18 ± 3.27	22.95 ± 3.09	0.53 (.590)
Depression	No	4,939 (87.0)	492 (8.7)	244 (4.3)	28.43 (< .001)	3,309 (81.4)	495 (12.2)	263 (6.5)	14.08 (.001)
	Yes	121 (73.8)	24 (14.6)	19 (11.6)		96 (70.6)	21 (15.4)	19 (14.0)	
Suicidal ideations	No	4,755 (87.6)	452 (8.3)	222 (4.1)	60.12 (< .001)	3,236 (82.1)	462 (11.7)	245 (6.2)	47.86 (< .001)
	Yes	305 (74.4)	64 (15.6)	41 (10.0)		170 (65.1)	54 (20.7)	37 (14.2)	

^a^*ADL* Activities of daily living, ^b^*IADL* Instrumental activities of daily living, ^c^*BMI* Body mass index

### Differences in fall frequency according to chronic disease characteristics

In both groups, recurrent falls rates were significantly associated with an impaired cognitive function, with higher number of chronic diseases, and higher number of prescribed medications. In the young-old group, recurrent falls rates were significantly associated with hypertension, stroke, angina and myocardial infarction, diabetes, arthritis, osteoporosis, lumbodynia, sciatic neuralgia, cataract, glaucoma, chronic kidney diseases, urinary incontinence, anemia, insomnia, and Parkinson’s disease. In the old-old group, recurrent falls rates were significantly associated with hypertension, arthritis, osteoporosis, lumbodynia, sciatic neuralgia, glaucoma, urinary incontinence, anemia, dementia, insomnia, and Parkinson’s disease (Table 3).

**Table 3:** Differences in fall frequency according to chronic disease characteristics (*N* = 10,043)

Variables	Category	Young-old (*n* = 5,838)				Old-old (*n* = 4,205)				
		No fall (*n* = 5,060)	Single fall (*n* = 516)	Recurrent fall (*n* = 263)	Χ^2^, F(*P*) Scheffe		No fall (*n* = 3,406)	Single fall (*n* = 516)	Recurrent fall (*n* = 283)	Χ^2^, F(*P*) Scheffe
Cognitive function		26.31 ± 3.04^a^	26.04 ± 3.07^b^	25.20 ± 3.89^c^	17.23 (< .001) a > b, c		23.94 ± 4.26^a^	23.15 ± 4.37^b^	23.49 ± 4.52^c^	8.46 (< .001) a > b, c
Number of chronic diseases		2.37 ± 1.74	3.01 ± 1.91	3.74 ± 2.25	98.29 (< .001)		2.91 ± 1.76	3.48 ± 1.86	3.92 ± 2.01	58.95 (< .001)
Number of prescribed medications		3.33 ± 3.16	4.42 ± 3.57	5.62 ± 4.25	83.06 (< .001)		4.20 ± 3.23	4.94 ± 3.56	5.68 ± 3.85	33.89 (< .001)
Hypertension	No	2,363 (88.7)	203 (7.6)	97 (3.6)	18.71 (< .001)		1,210 (83.3)	166 (11.4)	76 (5.2)	10.14 (.006)
	Yes	2,696 (84.9)	313 (9.9)	166 (5.2)			2,196 (79.8)	350 (12.7)	207 (7.5)	
Stroke	No	4,787 (87.0)	482 (8.8)	231 (4.2)	21.80 (< .001)		3,116 (81.3)	464 (12.1)	252 (6.6)	2.84 (.241)
	Yes	272 (80.5)	34 (10.1)	32 (9.5)			290 (77.7)	53 (14.2)	30 (8.0)	
Angina and myocardial infarction	No	4,750 (86.9)	479 (8.8)	236 (4.3)	7.70 (.021)		3,151 (81.3)	474 (12.2)	251 (6.5)	5.47 (.065)
	Yes	310 (82.9)	37 (9.9)	27 (7.2)			254 (77.4)	42 (12.8)	32 (9.8)	
Diabetes	No	3,963 (87.9)	371 (8.2)	174 (3.9)	30.17 (< .001)		2,596 (81.2)	393 (12.3)	209 (6.5)	0.81 (.668)
	Yes	1,096 (82.4)	145 (10.9)	89 (6.7)			810 (80.4)	123 (12.2)	74 (7.3)	
Arthritis	No	3,688 (88.6)	324 (7.8)	149 (3.6)	52.00 (< .001)		2,135 (83.5)	284 (11.1)	138 (5.4)	29.02 (< .001)
	Yes	1,372 (81.8)	192 (11.4)	114 (6.8)			1,270 (77.2)	232 (14.1)	144 (8.7)	
Osteoporosis	No	4,528 (87.9)	420 (8.2)	201 (3.9)	65.95 (< .001)		2,963 (82.7)	408 (11.4)	213 (5.9)	46.15 (< .001)
	Yes	532 (77.1)	96 (13.9)	62 (9.0)			442 (71.3)	109 (17.6)	69 (11.1)	
Lumbodynia and sciatic neuralgia	No	4,127 (88.3)	371 (7.9)	174 (3.7)	61.63 (< .001)		2,459 (83.3)	322 (10.9)	172 (5.8)	33.08 (< .001)
	Yes	933 (79.9)	145 (12.4)	90 (7.7)			946 (75.7)	194 (15.5)	110 (8.8)	
Cataract	No	4,775 (87.2)	468 (8.5)	236 (4.3)	18.91 (< .001)		3,131 (81.3)	468 (12.2)	250 (6.5)	4.17 (.124)
	Yes	285 (79.2)	48 (13.3)	27 (7.5)			275 (77.5)	48 (13.5)	32 (9.0)	
Glaucoma	No	4,968 (86.8)	500 (8.7)	253 (4.4)	10.14 (.006)		3,314 (81.2)	500 (12.3)	266 (6.5)	9.93 (.007)
	Yes	92 (77.3)	16 (13.4)	11 (9.2)			92 (73.6)	16 (12.8)	17 (13.6)	
Cancer	No	4,862 (86.7)	491 (8.8)	252 (4.5)	1.13 (.568)		3,288 (81.0)	498 (12.3)	273 (6.7)	0.01 (.998)
	Yes	197 (84.5)	25 (10.7)	11 (4.7)			118 (80.8)	18 (12.3)	10 (6.8)	
Chronic kidney diseases	No	4,982 (86.8)	508 (8.8)	251 (4.4)	14.28 (.001)		3,335 (81.1)	506 (12.3)	272 (6.6)	4.14 (.126)
	Yes	78 (80.4)	7 (7.2)	12 (12.4)			71 (77.2)	10 (10.9)	11 (12.0)	
Prostatic hypertrophy	No	4,727 (86.8)	482 (8.8)	239 (4.4)	2.60 (.272)		2,998 (80.9)	460 (12.4)	247 (6.7)	0.74 (.690)
	Yes	333 (85.2)	34 (8.7)	24 (6.1)			408 (81.6)	56 (11.2)	36 (7.2)	
Urinary incontinence	No	4,981 (86.9)	499 (8.7)	253 (4.4)	13.99 (.001)		3,310 (81.5)	495 (12.2)	258 (6.3)	29.84 (< .001)
	Yes	79 (74.5)	17 (16.0)	10 (9.4)			96 (67.6)	21 (14.8)	25 (17.6)	
Anemia	No	5,001 (86.8)	503 (8.7)	256 (4.4)	9.96 (.007)		3,309 (81.3)	495 (12.2)	265 (6.5)	11.61 (.003)
	Yes	59 (74.7)	13 (16.5)	7 (8.9)			97 (71.3)	21 (15.4)	18 (13.2)	
Dementia	No	5,026 (86.7)	508 (8.8)	260 (4.5)	5.22 (.074)		3,330 (81.2)	499 (12.2)	270 (6.6)	7.37 (.025)
	Yes	34 (75.6)	8 (17.8)	3 (6.7)			76 (71.7)	17 (16.0)	13 (12.3)	
Insomnia	No	4,918 (86.9)	498 (8.8)	243 (4.3)	19.86 (< .001)		3,280 (81.4)	486 (12.1)	264 (6.6)	10.26 (.006)
	Yes	141 (78.8)	18 (10.1)	20 (11.2)			125 (71.8)	30 (17.2)	19 (10.9)	
Parkinson's disease	No	5,037 (86.9)	506 (8.7)	255 (4.4)	42.45 (< .001)		3,376 (81.3)	505 (12.2)	269 (6.5)	36.56 (< .001)
	Yes	23 (54.8)	10 (23.8)	9 (21.4)			30 (54.5)	11 (20.0)	14 (25.5)	

### Factors affecting recurrent falls

The Hosmer–Lemeshow test showed that the fitness of the extracted models for the young-old and old-old elderly people was 0.77 and 0.32, respectively, indicating that the estimated models had a statistically good fit.

In the young-old group, those with limitations in ADL and who used visual aids were 2.25 and 1.56 times more likely to experience recurrent falls than those without such limitations. When the cognitive function score increased by one point, the odds of recurrent falls increased by 0.93 times. Those with suicidal ideations experienced recurrent falls 1.69 times more than those without suicidal ideations. As the number of chronic diseases and prescribed medications increased by one, the odds of recurrent falls increased by 1.22 and 1.06 times (Table 4).

**Table 4:** Factors that affect recurrent falls in the young-old elderly people

Variables	Young-old					
	B	SE	Wald	P	OR	95% CI
Intercept	− 2.53	0.50	25.76	< .001	0.08	-
Limitations in ADL^a^ (ref = none)	0.81	0.23	12.48	< .001	2.25	1.44–3.54
Visual aids (ref = none)	0.45	0.14	9.56	.002	1.56	1.18–20.7
Cognitive function	− 0.07	0.02	13.98	< .001	0.93	0.90–0.97
Suicidal ideations (ref = none)	0.53	0.19	7.84	.005	1.69	1.17–2.45
Number of chronic diseases	1.20	0.04	23.67	< .001	1.22	1.13–1.32
Number of prescribed medications	0.06	0.02	7.67	.006	1.06	1.02–1.11

^a^*ADL* Activities of daily living

In the old-old group, those who had no spouse, were NBLSS beneficiaries, did not exercise, had limitations in ADL, used visual aids, and had suicidal ideation were 0.76, 1.53, 0.68, 1.98, 1.54, and 1.63 times more likely to experience recurrent falls. As the number of chronic diseases increased by one, the odds of recurrent falls increased by 1.18 times, and those with Parkinson's disease had a 3.53 times increase in the odds of recurrent falls (Table 5).

**Table 5:** Factors that affect recurrent falls in the old-old elderly

Variables	Old-old					
	B	SE	Wald	*P*	OR	95% CI
Intercept	− 3.76	0.18	423.50	< .001	0.02	-
Having a spouse (ref = none)	− 0.28	0.13	4.48	.034	0.76	0.59–0.98
NBLSS^a^ beneficiary (ref = none)	0.42	0.19	5.00	.025	1.53	1.05–2.20
Exercise (ref = none)	-0.39	0.13	8.91	.003	0.68	0.53–0.88
Limitations in ADL^b^ (ref = none)	0.68	0.16	19.17	< .001	1.98	1.46–2.69
Visual aids (ref = none)	0.43	0.14	9.63	.002	1.54	1.17–2.02
Suicidal ideations (ref = none)	0.49	0.20	5.88	.015	1.63	1.10–2.43
Number of chronic diseases	0.17	0.03	25.03	< .001	1.18	1.11–1.26
'Parkinson’s disease (ref = none)	1.26	0.33	14.26	< .001	3.53	1.83–6.79

^a^*NBLSS* National Basic Livelihood Security System, ^b^*ADL* Activities of daily living

## Discussion

This study assessed the predictive factors of recurrent falls by age group in the young-old and old-old elderly. Both single and recurrent fall rates were higher in the old-old elderly, which is consistent with the results of a previous study that reported an increased rate of falls with age [5]. Additionally, half of those who experienced a single fall also experienced recurrent falls, which is consistent with the results reported by Kabeshova et al. [9].

The predictive factors of a high recurrence rate of falls were determined by focusing on the commonalities and differences between elderly people groups. In both young-old and old-old groups, factors that affected recurrent falls were limitations in ADL, use of visual aids, presence of suicidal ideations, and a number of chronic diseases. The young-old and old-old elderly people with limitations in ADL were more likely to experience recurrent falls, which is consistent with the results of a previous study [18] that showed that the risk of falls increased as the ability to perform ADL decreased. Elderly people have been shown to have balance difficulties in sitting to standing and surface-to-surface transfer [8]. Decreased body function may most likely lead to an increase in the rate of falls; thus, the physical functions of elderly people must be assessed to propose measures to prevent falls.

The young-old and old-old elderly people who use visual aids may experience more recurrent falls than those who do not use visual aids. Visual aids are required in those with a decreased visual function, and our finding on the relationship between the use of visual aids and falls are consistent with the findings of studies showing that sensory deficits such as vision and hearing impairments may be closely related to falls [19–21]. The WHO also suggested poor balance and limited vision as risk factors for falls. Therefore, creating an appropriate environment and developing auxiliary devices to compensate for decreased sensory functions may be necessary [3].

The young-old and old-old elderly people with suicidal ideations experienced recurrent falls more frequently than those without. Considering the close relationship between depression and suicidal thoughts, this finding was partially consistent with that of a study that showed a strong relationship between depression and falls [22]. In a study using large-scale national data [23], depression, along with the fear of falling, was identified as a risk factor for falls in the elderly. However, although both depression and suicidal ideations were assessed in our study, only suicidal ideations were shown to affect falls significantly. A previous study noted that depression affected single falls, whereas recurrent falls were affected by not only depression but also multiple factors including sleep disturbances and subjective stress [24]. In fact, in a study on the effects of suicidal thoughts in the elderly, it was observed that higher levels of depression, having no co-residing family members, and less active participation in social activities led to increased suicidal thoughts [25]. Since suicidal ideations reflect both family structures and social activities, multi-dimensional approaches at the individual, family, and community levels are necessary to prevent recurrent falls.

In our study, as the number of chronic diseases increased by one, the young-old and old-old elderly people were more likely to experience recurrent falls. This is consistent with the findings of a study that showed that the number of chronic diseases present was related to falls [5]. In another study [26], elderly people with chronic diseases believed that their subjective health was bad, which led to a vicious cycle of increased fear and falls. Therefore, interventions that consider the physical health and psychological aspects in the elderly people are necessary.

There were some differences in the factors that affected recurrent falls in the young-old and old-old elderly. In the young-old elderly, cognitive function and number of prescribed medications used were predictors of recurrent falls. This is consistent with the finding that decreased cognitive function increased the risk of falls [27]. In the young-old elderly, as cognitive function started to decline, there were also differences in the level of decrease in cognitive function, which may have affected the differences observed in the results of this study.

As the number of prescribed medications used increased by one, the odds of recurrent falls increased by 1.06 times, which is consistent with the finding that the use of medication increased the risk of falls in the elderly people [28]. Anti-convulsant, anti-Parkinson, anti-anxiety, sleeping, other anti-psychotic, anti-hypertensive, and cardiac medications, tranquilizers, and diuretics, which increase the risk of falls [29], are medications that are prescribed commonly to the elderly. It has been demonstrated that the use of an increased number of medications increases the likelihood of side effects, adverse drug reactions, and drug interactions in the elderly people [30–32]. Therefore, physicians must provide explanations for medications that greatly increase the risk of falls to the elderly, and education on behavior guidelines may be necessary to prevent falls.

Unique predictive factors of recurrent falls in the old-old elderly people were spouse status, being NBLSS beneficiaries, less exercise, and the presence of Parkinson’s disease. As noted in a study reporting that having a spouse was a factor that affected falls [7], our finding suggested that having a spouse affected both single and recurrent falls. The old-old elderly people are less influenced by their spouse at home because they participate less in work or social life. Thus, support systems that can continue to provide support, such as spouses, can prevent recurrent falls in those with reduced body functions. 

Furthermore, NBLSS beneficiaries were 1.53 times more likely to experience recurrent falls, which supported a WHO report that included socioeconomic factors, such as poverty, as risk factors for falls [3]. This finding suggested that NBLSS beneficiaries are socially and economically vulnerable, which may also indicate that the risk of falls in the residential environment is high. In the elderly, the risks of fall-related injuries and death are high because of aging-related physical, sensory, and cognitive changes and unsafe environments [3]. Since having a safe environment is an important factor for the prevention of falls, NBLSS beneficiaries may require support for the assessment and improvement of the residential environment for them to have a safe living environment.

Those who did not exercise were more likely to experience recurrent falls. This finding is consistent with the results of a previous study that indicated that a lack of exercise was the main risk factor for falls in the elderly people over the age of 80 years [5]. However, a previous study reported exercise time as a risk factor for falls in those over the age of 85 years. The risk of falls was higher in those who exercised for a short period of 10–20 min than in those who exercised for 30 min or more [33], which suggested that the type, intensity, and duration of exercise affected the risk of falls. Therefore, safety must be prioritized in exercise programs for the elderly, particularly the old-old elderly people. Because exercise is important for elderly people with weak physical functions, interventions that are safe for elderly people must be developed.

Lastly, those with Parkinson’s disease were more likely to experience recurrent falls than those without, which is consistent with previous results that indicated that Parkinson’s disease is closely related to falls because it affects motor function [34]. Moreover, falls are one of important factors causing disability in patients with Parkinson’s disease [35]. Therefore, these patients must be considered a high-risk group, and appropriate measures to prevent falls are necessary.

This study observed differences in factors related to recurrent falls between the young-old and old-old elderly. Risk assessments that reflect the characteristics of different age groups of elderly people must be developed to screen for recurrent falls in the community. Additionally, further studies that evaluate the effects of interventions and strategies that can prevent recurrent falls in high-risk groups are required.

The cross-sectional design of this study limited the assessment of causality of the relationship between the variables. It is recommended that longitudinal studies that include causal variables to assess the cause-and-effect relationship should be conducted in the future. Furthermore, fall experience and related factors were assessed using a self-reported questionnaire. Therefore, it would be necessary to perform objective measurement in future studies to compare with the results of subjective questionnaires.

## Conclusions

This study found that there is a difference in the factors related to recurrent falls between the young-old and old-old elderly. The results of this study indicate that it is necessary to implement an intervention program to prevent fall recurrence by age group in consideration of the risk factors for fall recurrence in each elderly people group.

## Supplementary Information

**Additional file 1.** 2017 National Survey of Older Koreans_FALL.

## Data Availability

The datasets analyzed during the current study are available from the Health and Welfare Data Portal and open to all researchers. The full dataset are available from the authors upon request on the Health and Welfare Data Portal repository (https://data.kihasa.re.kr/kihasa/kor/contents/ContentsList.html), [accession number: 5_National Survey of Older Koreans; accession year: 2017]. The English dataset used during the current study are included in the supplementary information files (2017 National Survey of Older Koreans_FALL.pdf). English data used in this study are available in Appendix 1.
